# Innate Immune Response of Human Plasmacytoid Dendritic Cells to Poxvirus Infection Is Subverted by Vaccinia E3 via Its Z-DNA/RNA Binding Domain

**DOI:** 10.1371/journal.pone.0036823

**Published:** 2012-05-14

**Authors:** Hua Cao, Peihong Dai, Weiyi Wang, Hao Li, Jianda Yuan, Fangjin Wang, Chee-Mun Fang, Paula M Pitha, Jia Liu, Richard C Condit, Grant McFadden, Taha Merghoub, Alan N Houghton, James W Young, Stewart Shuman, Liang Deng

**Affiliations:** 1 Dermatology Service, Department of Medicine, Memorial Sloan-Kettering Cancer Center, New York, New York, United States of America; 2 Molecular Biology Program, Sloan-Kettering Institute, Memorial Sloan-Kettering Cancer Center, New York, New York, United States of America; 3 Immune Monitoring Core Facility, Ludwig Center for Cancer Immunotherapy, Memorial Sloan-Kettering Cancer Center, New York, New York, United States of America; 4 Laboratory of Cellular Immunobiology, Adult Allogenic Bone Marrow Transplantation Service, Division of Hematologic Oncology, Department of Medicine, Memorial Sloan-Kettering Cancer Center, New York, New York, United States of America; 5 Immunology Program, Sloan-Kettering Institute, Memorial Sloan-Kettering Cancer Center, New York, New York, United States of America; 6 Lucille Castori Center for Microbes, Inflammation and Cancer, Memorial Sloan-Kettering Cancer Center, New York, New York, United States of America; 7 Department of Biology, The Johns Hopkins University, Baltimore, Maryland, United States of America; 8 Department of Molecular Genetics and Microbiology, College of Medicine, University of Florida, Gainesville, Florida, United States of America; Kantonal Hospital St. Gallen, Switzerland

## Abstract

Plasmacytoid dendritic cells (pDCs) play important roles in antiviral innate immunity by producing type I interferon (IFN). In this study, we assess the immune responses of primary human pDCs to two poxviruses, vaccinia and myxoma virus. Vaccinia, an *orthopoxvirus*, was used for immunization against smallpox, a contagious human disease with high mortality. Myxoma virus, a *Leporipoxvirus*, causes lethal disease in rabbits, but is non-pathogenic in humans. We report that myxoma virus infection of human pDCs induces IFN-α and TNF production, whereas vaccinia infection does not. Co-infection of pDCs with myxoma virus plus vaccinia blocks myxoma induction effects. We find that heat-inactivated vaccinia (Heat-VAC; by incubating the virus at 55°C for 1 h) gains the ability to induce IFN-α and TNF in primary human pDCs. Induction of IFN-α in pDCs by myxoma virus or Heat-VAC is blocked by chloroquine, which inhibits endosomal acidification required for TLR7/9 signaling, and by inhibitors of cellular kinases PI3K and Akt. Using purified pDCs from genetic knockout mice, we demonstrate that Heat-VAC-induced type I IFN production in pDCs requires the endosomal RNA sensor TLR7 and its adaptor MyD88, transcription factor IRF7 and the type I IFN feedback loop mediated by IFNAR1. These results indicate that (i) vaccinia virus, but not myxoma virus, expresses inhibitor(s) of the poxvirus sensing pathway(s) in pDCs; and (ii) Heat-VAC infection fails to produce inhibitor(s) but rather produces novel activator(s), likely viral RNA transcripts that are sensed by the TLR7/MyD88 pathway. Using vaccinia gene deletion mutants, we show that the Z-DNA/RNA binding domain at the N-terminus of the vaccinia immunomodulatory E3 protein is an antagonist of the innate immune response of human pDCs to poxvirus infection and TLR agonists. The myxoma virus ortholog of vaccinia E3 (M029) lacks the N-terminal Z-DNA/RNA binding domain, which might contribute to the immunostimulating properties of myxoma virus.

## Introduction

Induction of antiviral effectors like type I interferon (IFN) in a nonpermissive host underlies one mechanism that restricts poxvirus host tropism 1. The interactions of poxviruses with the sentinel cells of the host immune system, particularly with plasmacytoid dendritic cells (pDCs), are of significance because: (i) pDCs are potent producers of type I IFN during virus infections 2; (ii) through the production of type I IFN, pDCs activate NK cells, conventional DCs, Bcells, and T cells to augment antiviral innate and adaptive immunity 3; and (iii) type I IFN signaling is crucial for protection of mice against infection by vaccinia virus 4 or myxoma virus 5.

pDCs can sense virus infections through the recognition of viral RNA by TLR7 and viral DNA by TLR9. TLR7 and TLR9 localize within endosomes and require endosomal acidification and maturation to signal through their common adaptor MyD88 6,7. Following the engagement of TLR7/TLR9 and MyD88, a multi-protein complex is formed, leading to the phosphorylation, activation, and nuclear translocation of transcription factor IRF7, which induces type I IFN production 3,8–10. Type I IFNs bind to the IFN-α/β receptor and induce antiviral states in many cell types through the expression and activation of effectors such as protein kinase R, 2′-5′ oligoadenylate synthetase, and RNase L 11.

Poxviruses are large cytoplasmic dsDNA viruses that can manipulate many of the host immune pathways 12. Vaccinia, a prototypal *Orthopoxvirus*, has been extensively used to vaccinate against human smallpox. Despite its successes as a vaccine, severe complications of smallpox vaccination can occur, including eczema vaccinatum in people with atopic dermatitis and progressive vaccinia in immunocompromised hosts. Myxoma virus belongs to the *Leporipoxvirus* genus and causes lethal myxomatosis in European rabbits. Myxoma virus infection is rabbit-specific and the virus is nonpathogenic in mice and humans 13. We hypothesize that myxoma virus and vaccinia are sensed differently and trigger different immune responses in infected innate sentinel cells, such as pDCs, that might contribute to their recognition by early immune response pathways, and thus affect their pathogenesis and immunogenicity in humans.

How poxviruses are sensed or evade sensing by innate immune cells such as pDCs is not very well understood. Ectromelia virus, the causative agent of mousepox, induces IFN-α production in murine pDCs through a mechanism that at least partly depends on TLR9, such that mice lacking TLR9 are more susceptible to ectromelia infection 14. We recently reported that myxoma virus infection of murine pDCs induces type I IFN via a signaling pathway involving TLR9/MyD88, IRF5/IRF7 and IFNAR 15. Here, we show that myxoma infection of primary human pDCs induces the production of IFN-α and TNF. Myxoma induction of IFN-α and TNF can be blocked by chloroquine, which inhibits endosomal acidification and maturation, and by inhibitors of cellular protein kinases PI3K and Akt. These results indicate that myxoma virus infection in human pDCs is sensed through an endosomal TLR, PI3K/Akt-dependent signaling pathway. We also show that vaccinia infection of human pDCs strongly inhibits IFN-α and TNF induction by myxoma virus and by agonists of TLR7/9.

To explore the mechanisms through which vaccinia might block its sensing by human pDCs, we tested whether Heat-VAC stimulates human pDCs. It had been reported previously that incubating vaccinia at 55°C for 1 h renders the virus capable of activating human monocyte-derived conventional DCs 16. We find that Heat-VAC enters pDCs through its classical entry-fusion pathway and induces pDCs to produce IFN-α and TNF. Using purified pDCs from Flt3L-cultured bone marrow-derived dendritic cells (Flt3L-BMDCs) from various knock-out (KO) mice, we show that Heat-VAC-induced type I IFN production is dependent on the endosomal RNA sensor TLR7 and its adaptor MyD88, the transcription factor IRF7 and IFNAR1 which mediates the type I IFN positive feedback loop.

Finally, we addressed whether vaccinia E3, a key immunomodulatory protein 17 that binds Z-DNA/RNA via a specific domain at its N-terminus, and dsRNA via a distinct C-terminal domain, plays a role in mediating the inhibitory effects. We find that whereas co-infection with wild-type (WT) vaccinia or E3LΔ26C virus (in which the E3 C-terminal dsRNA binding domain is deleted) significantly attenuated the induction of IFN-α and TNF by myxoma virus or Heat-VAC, co-infection with vaccinia mutant ΔE3L (E3 null) or E3LΔ83N (in which the E3 N-terminal Z-DNA/RNA binding domain is deleted) only partially reduced IFN-α and TNF induction. Our results reveal a new aspect of the innate immune evasion strategy of vaccinia virus in human pDCs, with implications for the exploitation of poxviruses for therapeutic or vaccination purposes.

## Results

### Myxoma Virus Infection Induces Ifn-α And Tnf Production In Human Pdcs

**Figure 1:**
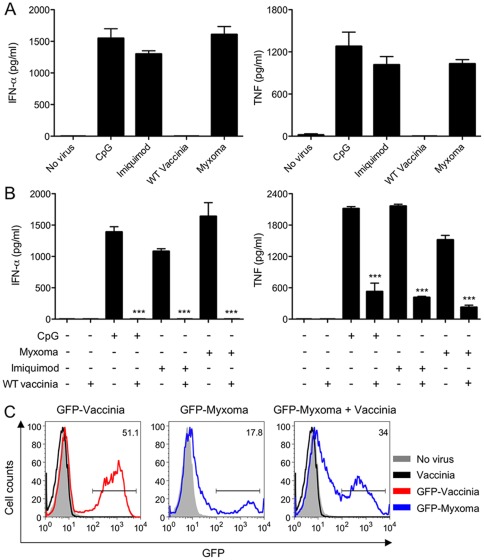
Myxoma virus infection induces IFN-α and TNF production in human pDCs. (A) Freshly isolated pDCs (2×10^5^) were stimulated with CpG2216 (10 μg/ml) or imiquimod (5 μg/ml), or infected with vaccinia or myxoma virus at a multiplicity of 10 (MOI = 10). The concentrations of IFN-α and TNF in the culture supernatants collected at 20 h post treatment were determined by ELISA. The values shown are averages of triplicate means (± SEM) of three independent experiments using human pDCs isolated from three different donors. (B) pDCs were infected with vaccinia followed by addition of CpG2216 (10 μg/ml) or imiquimod (5 μg/ml), or co-infected with vaccinia plus myxoma virus at a MOI of 10 for each virus. Control cells that were treated with CpG or imiquimod, or infected singly with vaccinia or myxoma virus were included. The concentrations of IFN-α and TNF in the culture supernatants collected at 20 h post treatment were determined by ELISA. The values shown are averages of triplicate means (± SEM) of three independent experiments using human pDCs isolated from three different donors (***, *p*<0.001). (C) Freshly isolated human pDCs were infected with WT vaccinia, GFP-expressing vaccinia or myxoma (GFP-Vaccinia or GFP-Myxoma) alone at a MOI of 10, or co-infected with WT vaccinia plus GFP-Myxoma. GFP expression in infected pDCs (CD123^+^BDCA2^+^ cells) was determined by FACS. The experiments were repeated twice. The results of a representative are shown.

To test whether primary human pDCs respond differently to vaccinia (an *Orthopoxvirus* that is potentially pathogenic in humans) and myxoma virus (a *Leporipoxvirus* that is non-pathogenic in humans), we purified pDCs from human peripheral blood mononuclear cells using anti-BDCA-4 antibody-coated magnetic beads. The resulting pDC-enriched preparations (CD123^+^/BDCA2^+^ cells) had a purity of 60–80% as assessed by flow cytometry (data not shown). Treatment of pDCs with either TLR9 agonist CpG or TLR7 agonist imiquimod co-induced the production and secretion of IFN-α and TNF (Fig. 1A). Infection of pDCs with myxoma virus also induced the production of comparable levels of IFN-α and TNF (Fig. 1A). By contrast, pDCs did not secrete IFN-α or TNF when infected with vaccinia virus (Fig. 1A).

### Vaccinia Virus Down-Regulates Cytokine Induction By Either Cpg Or Myxoma Virus In Human Pdcs

We hypothesized that vaccinia virus produces inhibitor(s) of type I IFN and TNF induction in pDCs. To test this idea, purified pDCs were either: (i) treated with CpG or imiquimod; (ii) infected with myxoma virus alone; (iii) infected with vaccinia followed by addition of CpG or imiquimod; or (iv) co-infected with vaccinia and myxoma virus. Supernatants were collected at 20 h post-treatment and assayed for IFN-α and TNF production. We found that vaccinia infection of pDCs completely blocked the induction of IFN-α in response to myxoma virus, CpG or imiquimod (Fig. 1B). Vaccinia also inhibited the induction of TNF by myxoma virus, CpG, and imiquimod, but only by 86%, 75% and 78%, respectively (Fig. 1B). IFN-α production/secretion is therefore more sensitive to inhibition by vaccinia than is TNF production/secretion. These results in human pDCs are consistent with that from highly purified murine pDCs. We found that WT vaccinia infection had a stronger inhibitory effects on IFN-α/β than TNF 15. We suspect that could be due to differences in the regulatory pathways leading to the induction of TNF and IFN in pDCs.

Neither myxoma virus nor vaccinia infection of human pDCs was productive, i.e., cells infected at a multiplicity of 5 supported no increase in viral titers at 48 h post infection, but virus entry and early viral gene expression occurred in each case, as judged by the presence of green fluorescence in human pDCs infected with recombinant myxoma virus or vaccinia virus expressing green fluorescent protein (GFP) under the control of the vaccinia synthetic early/late promoter (Fig. 1C). In GFP-Vaccinia infected pDCs, 50% of cells were GFP-positive, whereas in GFP-Myxoma infected pDCs, only 18% of cells were positive for GFP. WT vaccinia virus was used as a negative control and no GFP signal was detected as expected. This result is similar to what we observed with purified murine pDCs 15. The apparent difference in infectivity could be due to differences in restricting the life cycle of vaccinia and myxoma virus by infected pDCs. Interestingly, co-infection of GFP-Myxoma and WT vaccinia leads to an increased number of GFP-positive cells (from 18% in GFP-Myxoma infection alone to 34% in co-infection), which is consistent with the notion that type I IFN signaling restricts viral life cycle 5. As shown in Fig. 1B, co-infection of myxoma and vaccinia leads to the attenuation of type I IFN production.

### Myxoma Virus Induction Of Ifn-α And Tnf In Human Pdcs Is Inhibited By Chloroquine

**Figure 2:**
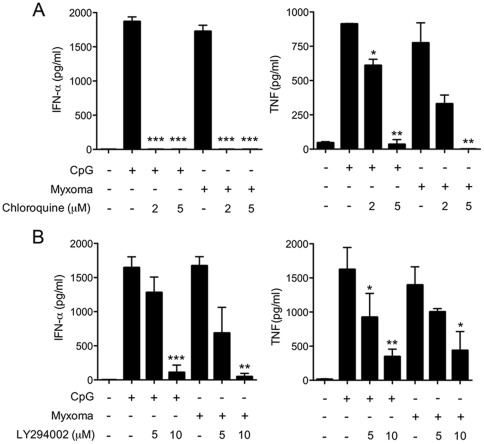
Chloroquine and PI3K inhibitor block the induction of IFN-α and TNF in human pDCs by myxoma virus. pDCs (2×10^5^) were stimulated with CpG2216 (10 μg/ml), or infected with myxoma virus (MOI = 10), and were then treated with or without inhibitors including chloroquine (A), and LY294002 (B) at indicated concentrations. Supernatants were collected at 20 h post treatment and measured for IFN-α and TNF concentrations by ELISA. The values shown are averages of triplicate means (± SEM) of three independent experiments using human pDCs isolated from three different donors (*, *p*<0.05; **, *p*<0.01; ***, *p*<0.001).

pDCs utilize TLR7 and TLR9 to detect viral nucleic acids and initiate an antiviral response. TLR9 has been implicated in recognizing viral DNA, as demonstrated for herpes simplex virus [Bibr pone.0036823-Lund1] [Bibr pone.0036823-Hochrein1] 18-20. pDCs rely on TLR7 in sensing RNA virus infection 21,22. Myxoma virus is sensed by TLR9/MyD88 in murine pDCs 15, whereas myxoma virus infection induces both type I IFN and TNF in primary human macrophages by a RIG-I-dependent sensing mechanism 23. Here we tested whether chloroquine, an inhibitor of endosomal acidification and maturation 21, would affect the innate responses of human pDCs to myxoma virus infection. Treatment of pDCs at 1 h post-inoculation with 2 µM and 5 µM chloroquine blocked IFN-αproduction, while reducing TNF production by 57% and 99%, respectively (Fig. 2A). Induction of IFN-α secretion by TLR9 agonist CpG was also blocked by 2 µM and 5 µM chloroquine, while CpG-induced TNF production was reduced by 33% and 96%, respectively (Fig. 2A). Imiquimod-induced IFN-α and TNF production was also similarly inhibited in the presence of chloroquine (data not shown). The greater sensitivity of IFN-α versus TNF induction to chloroquine inhibition could be related to the spatial and temporal regulation of IFN-α and TNF in early and late endosomes, respectively 24. These data implicate that endosomal acidification, such as that required for TLR9 signaling, is important for myxoma virus sensing by human pDCs.

### Pi3k/akt-Dependent Induction Of Ifn-α And Tnf In Human Pdcs By Myxoma Virus

Phosphoinositide 3-kinase (PI3K) has been implicated in diverse biological processes, including immune regulation 25. PI3K catalyzes the conversion of PtdIns(4,5)P2 to PtdIns(3,4,5)P3, an important second messenger. Recent studies have shown that PI3K is involved in both positive and negative regulation of TLR signaling 26. In human pDCs, PI3K activation is essential for type I IFN induction by CpG, herpes simplex virus, or influenza virus 27. To investigate if PI3K activity is required for the induction of IFN-α and TNF by myoxma virus, we infected pDCs for 1 h, then washed the cells and treated them with PI3K inhibitor LY294002 (LY). We found that treatment of myxoma-infected pDCs with 10 µM LY resulted in 97% inhibition of IFN-α secretion (Fig. 2B) and a 75% decrement in TNF production (Fig. 2B). Similar inhibitory effects were observed with CpG treated pDCs (Fig. 2B).

**Figure 3:**
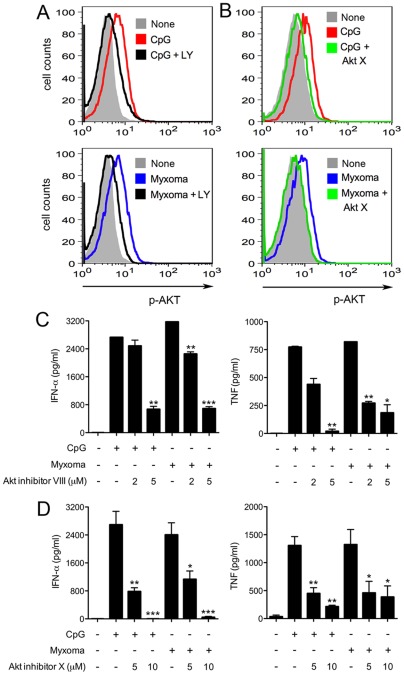
Akt inhibitors VIII and X block the induction of IFN-α and TNF in human pDCs by myxoma virus. (A, B) Human pDCs were cultured with CpG2216 (10 μg/ml), or infected with myxoma virus (MOI = 10), and were then treated with or without LY294002 (10 μm) or Akt inhibitor X (10 μm) for 90 min (CpG) or 8 h (Myxoma). Cells were stained with Alexa Fluor 647 anti-human AKT antibody that recognizes phospho-S473, and analyzed by flow cytometry. The results shown are representative of three separate experiments. (C, D) pDCs (2×10^5^)were stimulated with CpG2216 (10 μg/ml), or infected with myxoma virus (MOI = 10), and were then treated with or without Akt inhibitors VIII (C), or X (D) at indicated concentrations. Supernatants were collected at 20 h post treatment and measured for IFN-α and TNF concentrations by ELISA. The values shown are averages of triplicate means (± SEM) of three independent experiments using human pDCs isolated from three different donors (*, *p*<0.05; **, *p*<0.01; ***, *p*<0.001).

Akt (protein kinase B), a serine/threonine kinase and a downstream target of PI3K, is a regulator of cell metabolism, survival, and proliferation 28. PI3K generates PtdIns(3,4,5)P3, which recruits inactive Akt in the cytosol to the plasma membrane. The binding of PtdIns(3,4,5)P3 to the N-terminal pleckstrin homology (PH) domain of Akt allows phosphorylation of threonine-308 at the activation loop of the AKT kinase domain by 3-phosphoinositide-dependent protein kinase-1 (PDK-1). The activity of PDK-1 is also dependent on the binding of PtdIns(3,4,5)P3. Subsequent phosphorylation occurs at serine-473 in the hydrophobic regulatory domain by the mTORC2 complex, which is required for the activation of Akt 29. Guiducci et al. 27 showed that CpG treatment or infection with influenza virus induces Akt phosphorylation at Ser473 in pDCs. This induction can be inhibited by PI3K inhibitor LY. We observed that myxoma virus induction of Akt phosphorylation (p-AKT) at Ser473 occurs at 8 h post infection, as determined by intracellular staining with anti-p-AKT antibody against phospho-Ser473 followed by FACS analysis (Fig. 3A). LY inhibited both CpG- and myxoma-induced Akt phosphorylation in human pDCs (Fig. 3A).

To test if Akt kinase activity was required for IFN-α and TNF induction, we used two Akt inhibitors. Akt inhibitor VIII, a quinoxaline compound, inhibits Akt activity in a PH domain-dependent manner. It locks the enzyme in an inactive conformation through binding to two different functional regions 30. By contrast, Akt inhibitor X action is PH domain-independent. A phenoxazine derivative, Akt inhibitor X inhibits Akt phosphorylation and its kinase activity *in vitro* with minimum effect on PI3K and PDK-1. The exact mechanism of action of Akt inhibitor X is currently unknown 31. To avoid potential effects of Akt inhibitors on viral entry or uptake of TLR9 agonist CpG, we infected human pDCs with myxoma virus or treated them with CpG for 1 h prior to the addition of the inhibitors. We found that Akt inhibitors VIII and X partially attenuated IFN-α and TNF production by myxoma-infected pDCs in a dose-dependent manner (Fig. 3C and D). 5 µM Akt inhibitor VIII reduced IFN-α and TNF secretion by 78% and 77%, respectively (Fig. 3A). 10 µM Akt inhibitor X reduced IFN-a and TNF secretion by 98% and 65%, respectively. Similar inhibition was observed for CpG-induced production of IFN-α and TNF (Fig. 3C and D). In addition, Akt phosphorylation induced by CpG treatment or myxoma virus infection was inhibited in the presence of Akt inhibitor X (Fig. 3B). These results indicate that the PI3K/Akt pathway plays an important role in both the TLR9- and myxoma-triggered immune responses in human pDCs.

### Heat-Vac Induces Ifn-α And Tnf Production In Human Pdcs

**Figure 4:**
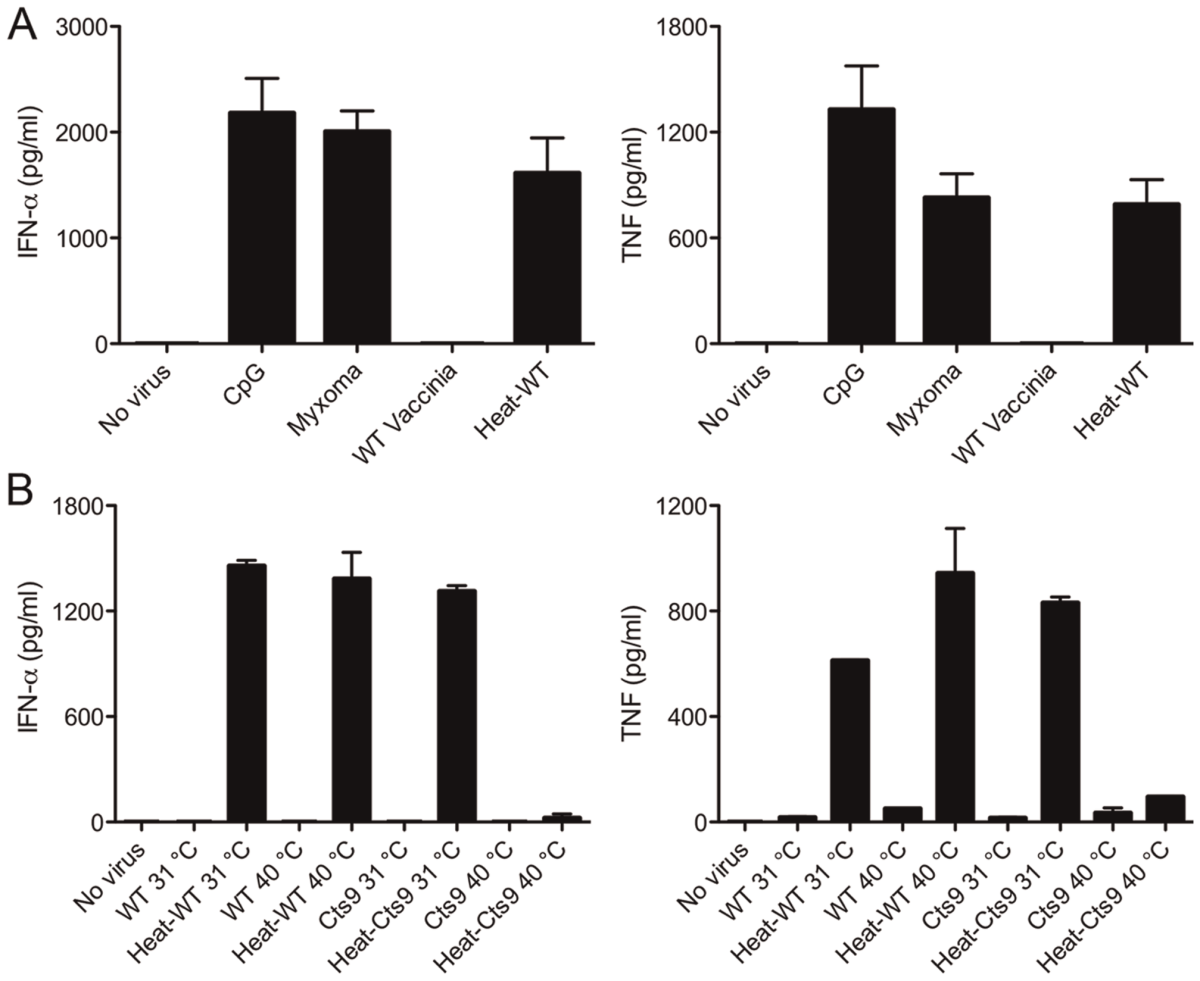
Heat-inactivated vaccinia infection induces IFN-α and TNF production in human pDCs. (A) Human pDCs (2×10^5^) were left untreated, or treated with CpG, or infected with myxoma virus, live vaccinia, or heat-inactivated vaccinia (55°C, 1 h). Supernatants were collected at 20 h post treatment and measured for IFN-α and TNF concentrations by ELISA. The values shown are averages of triplicate means (± SEM) of four independent experiments using human pDCs isolated from four different donors. (B) pDCs were infected as indicated with WT vaccinia grown in BSC40 cells at 31°C or 40°C (WT 31°C; WT 40°C; MOI = 10), or an equivalent amount of WT vaccinia heated at 55°C for 1 h (Heat-WT 31°C; Heat-WT 40°C), vaccinia mutant Cts9 grown in BSC40 cells at 31°C or 40°C (Cts9 31°C; Cts9 40°C; MOI = 10), or an equivalent amount of Cts9 heated at 55°C for 1 h (Heat-Cts9 31°C; Heat-Cts9 40°C). Supernatants were collected at 20 h post infection and measured for IFN-α and TNF concentrations by ELISA. The values shown are averages of triplicate means (± SEM) of three independent experiments using human pDCs isolated from three different donors.

Drillien et al. 16 reported that incubation of vaccinia at 55°C for 1 h rendered the virus essentially noninfectious but capable of activating human monocyte-derived dendritic cells, as demonstrated by upregulation of the co-stimulatory molecule CD86. Here we tested whether Heat-VAC can induce an innate cytokine response in human pDCs. Incubation of vaccinia at 55°C for 1 h decreased infectivity by 1000-fold, as determined by titration of plaque forming units on permissive BSC40 cell monolayers. We infected human pDCs with vaccinia at a multiplicity of 10, or with an equivalent amount of Heat-VAC. Myxoma virus infection and CpG treatment provided positive controls. We found that whereas untreated vaccinia failed to activate pDCs, Heat-VAC induced IFN-α and TNF production to levels similar to those induced by myxoma virus (Fig. 4A). Heating vaccinia at higher temperatures (65°C for 1 h or 100°C for 5 min) abolished the induction of IFN-α and TNF (data not shown). To understand the effects of heat-inactivation on viral gene expression, we assessed GFP expression at 6 h post infection using FACS analysis in human pDCs infected by heat-inactivated (at 55°C for 1 h) recombinant vaccinia expressing GFP under the vaccinia p7.5 promoter. We found that GFP expression was significantly reduced with heat-inactivated GFP-VAC (data not shown). This result indicates that Heat-VAC fails to produce viral proteins during infection in pDCs.

### Entry Of Heat-Vac Through The Poxvirus Fusion Complex Is Essential For Induction Of Ifn-α In Human Pdcs

We considered several possibilities to account for the inductive effects of heat-inactivated vaccinia: (i) heat-treatment liberates an inducing factor from the virion that triggers IFN-α and TNF production, whether or not the heated particles are taken up by the pDCs; (ii) heat-inactivated viral particles are taken up by pDCs and generate inducing substances intracellularly that are not normally present during vaccinia infection; or (iii) Heat-VAC infection produces inducer(s) present during normal infection with vaccinia, but fail to generate inhibitor(s) of innate immune signaling in pDCs. We first addressed the issue of virion uptake by pDCs.

Vaccinia virus enters the host cells through an entry-fusion complex composed of multiple virus-encoded proteins, including A28 32. To test whether Heat-VAC enters pDCs through this entry-fusion complex in order to trigger an innate immune response, we used a temperature-sensitive virus, Cts9. This mutant has a 2-bp deletion in the A28 gene, resulting in a truncated protein lacking 14 amino acids at the C-terminus 33. Mature virions of Cts9 produced at a permissive temperature (31°C) are infectious, whereas Cts9 virions produced at a non-permissive temperature (40°C) bind to cells but fail to enter 33. In the experiments shown in Fig. 4C, we infected pDCs with WT or Cts9 viruses that had been grown in BSC40 cells at 31°C or 40°C and then purified by sedimentation through a sucrose gradient 33. pDCs were inoculated with equivalent virion aliquots (determined by *A*_260_) corresponding to a multiplicity of 10 for WT vaccinia or Cts9 grown at permissive temperature, and in parallel with aliquots of virions that were treated at 55°C for 1 h. We found that heat-inactivated WT vaccinia grown at either 31°C or 40°C, and heat-inactivated Cts9 grown at 31°C induced similar levels of IFN-α and TNF secretion. On the other hand, heat-inactivated Cts9 produced at 40°C failed to induce IFN-α and induced TNF to only 12% of the level induced by Cts9 produced at 31°C (Fig. 4B). This result indicates that Heat-VAC enters pDCs through an A28-dependent fusion mechanism to induce an innate cytokine-mediated immune response in human pDCs.

### Induction Of Ifn-α And Tnf By Heat-Vac Is Inhibited By Chloroquine, Pi3k Inhibitor Ly294002 And Akt Inhibitors Viii And X

**Figure 5:**
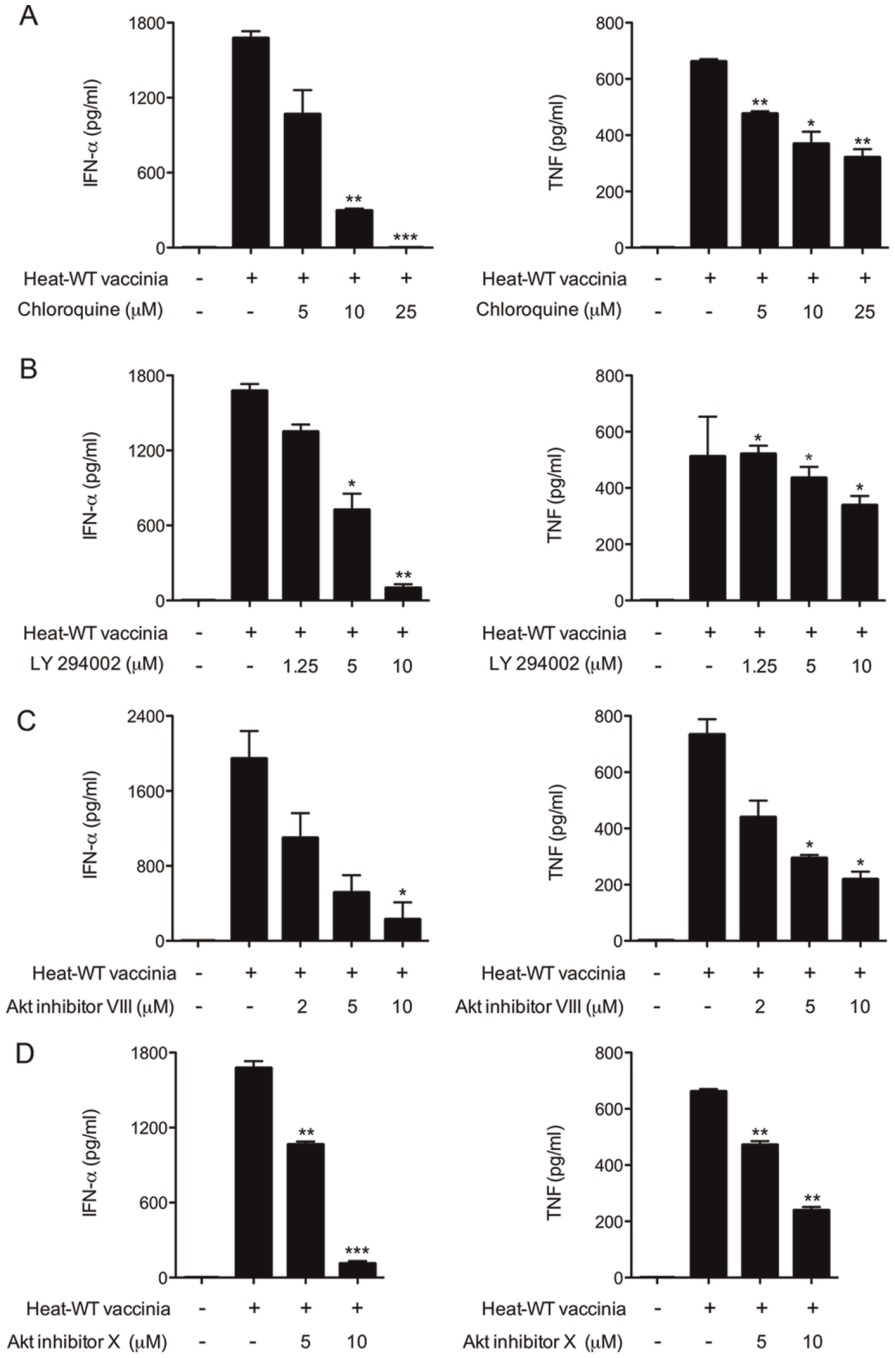
Chloroquine, PI3K and Akt inhibitors block the induction of IFN-α and TNF in human pDCs by heat-inactivated vaccinia virus. Human pDCs (2×10^5^) were infected as indicated with heat-inactivated vaccinia for 1 h. Cells were washed and incubated in fresh medium in the presence or absence of increasing concentrations of chloroquine (A), LY294002 (B), Akt inhibitor VIII (C), or Akt inhibitor X (D). Supernatants were collected at 20 h post treatment and assayed for IFN-α and TNF concentrations. The values shown are averages of triplicate means (± SEM) of three independent experiments using human pDCs isolated from three different donors (*, *p*<0.05; **, *p*<0.01; ***, *p*<0.001).

We next asked if Heat-VAC induces an antiviral response in pDCs via a similar pathway triggered by myxoma virus. We addressed this issue with the battery of small molecule inhibitors discussed above. First, we observed that chloroquine reduced IFN-α and TNF production by pDCs infected with heat-inactivated vaccinia in a dose-dependent fashion: 25 µM chloroquine completely blocked the production of IFN-α and reduced TNF level by 52% (Fig. 5A). By comparison, as little as 2 µM chloroquine completely blocked IFN-α production and reduced TNF secretion by 55% in myxoma-infected pDCs (Fig. 2A). Therefore, the induction of IFN-α and TNF by Heat-VAC is at least 10-fold less sensitive to chloroquine inhibition than is induction by myxoma virus infection. 10 µM PI3K inhibitor LY294002 inhibited IFN-α and TNF production by 93% and 33%, respectively in pDCs infected with Heat-VAC (Fig. 5B). 10 µM Akt inhibitor VIII inhibited IFN-α and TNF production by 89% and 71%, respectively (Fig. 5C); and 10 µM of Akt X reduced IFN-α and TNF production by 93% and 64%, respectively (Fig. 5D). These results indicate that Heat-VAC is sensed by pDCs through a pathway that is similar, but not identical, to that for detecting myxoma virus.

#### Tlr7 And Myd88 Are Required For The Induction Of Type I Ifn In Murine Pdcs By Heat-Vac

**Figure 6:**
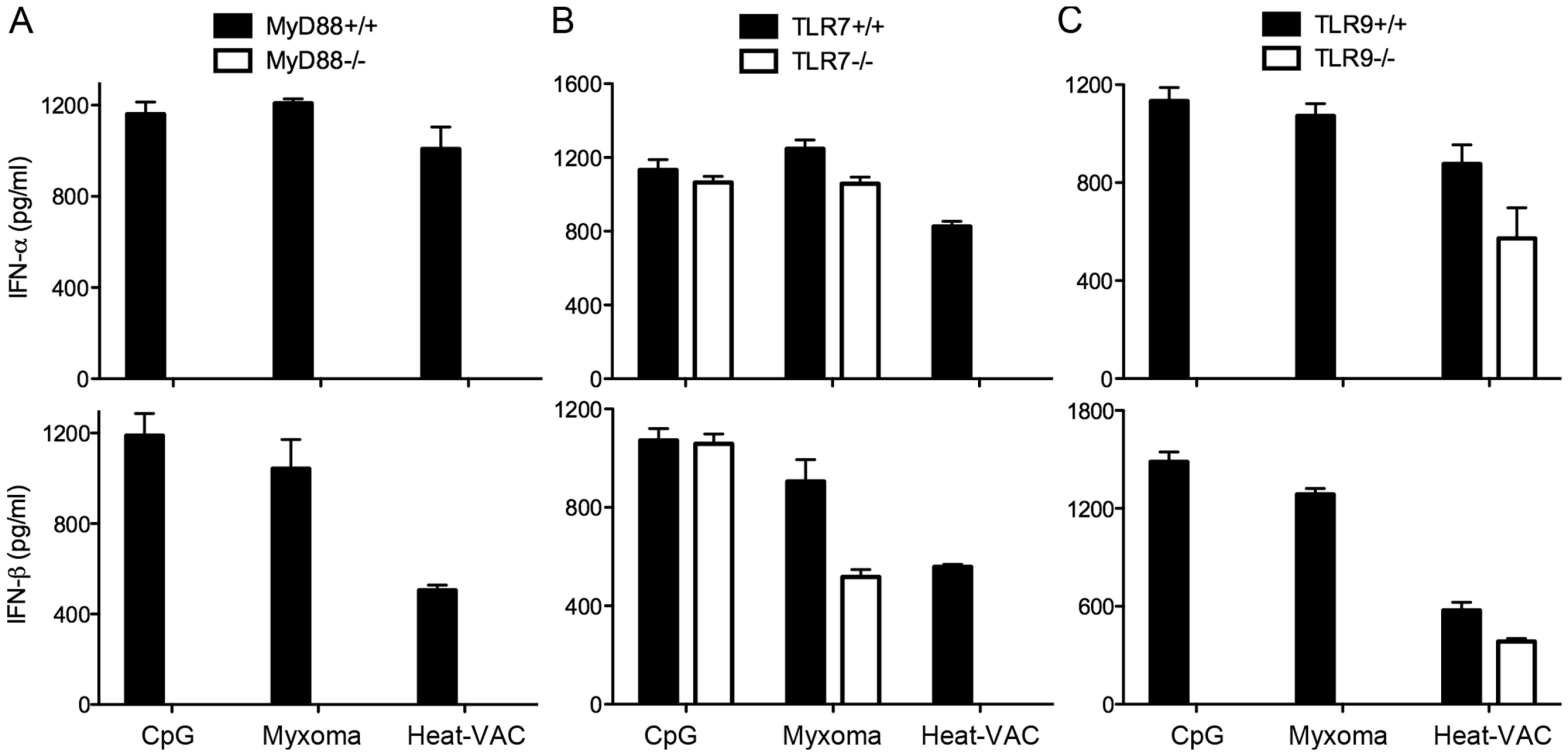
TLR7 and MyD88 are required for the induction of type I IFN by Heat-VAC in murine pDCs. Purified murine pDCs were obtained using FACS from Flt3L-BMDCs generated from MyD88^−/−^ (A), TLR7^−/−^ (B), TLR9^−/−^ (C) mice and age-matched WT controls. pDCs (2×10^5^) were stimulated with CpG, or infected with myxoma virus at a MOI of 10, or with an equivalent amount of Heat-VAC. Supernatants were collected at 22 h post infection. The concentrations of IFN-α/β were determined by ELISA. Data are means ± SEM. The combined results of three independently performed experiments are shown.

We took advantage of the murine genetic system to determine the mechanism of induction of type I IFN in pDCs by Heat-VAC. We purified pDCs from Flt3L-BMDCs from MyD88^−/−^, TLR7^−/−^, TLR9^−/−^ or age-matched WT control mice by FACS as described 15. The isolated pDCs are CD11c^+^B220^+^PDCA-1^+^, with a purity of greater than 98%. They were treated with CpG, or infected with myxoma virus at a MOI of 10, or with an equivalent amount of Heat-VAC. Supernatants were collected at 22 h post infection. The level of IFN-α/β was determined by ELISA. We found that Heat-VAC-induced production of IFN-α/β was abolished in MyD88^−/−^ or TLR7^−/−^ pDCs, but only modestly reduced in TLR9^−/−^ pDCs (Fig. 6A, B, and C). In contrast, myxoma-induced type I IFN induction was abolished in MyD88^−/−^, or TLR9^−/−^ pDCs, but modestly reduced in TLR7^−/−^ pDCs as reported previously (Fig. , 15). As a control, CpG induced type I IFN is abolished in TLR9^−/−^ or MyD88^−/−^ pDCs, but not affected in TLR7^−/−^ pDCs (Fig. 6). Taken together, these results indicate that Heat-VAC infection of pDCs leads to the production of RNA species that are detected by the endosomal RNA sensing pathway mediated by TLR7/MyD88.

#### Heat-Vac Induction Of Ifn-α/β Requires Irf7 And Ifnar1

**Figure 7:**
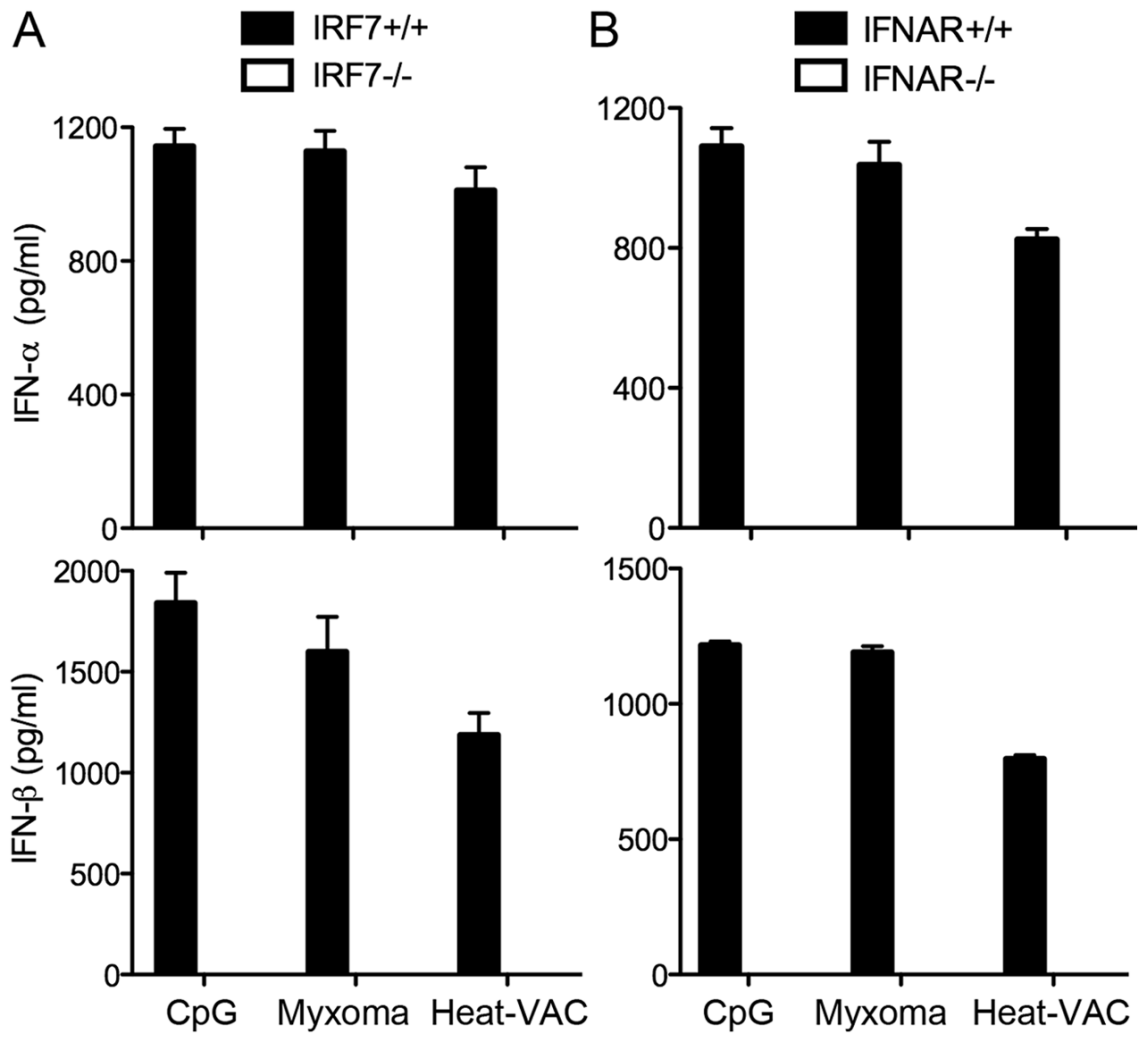
Heat-VAC induced production of type I IFN is dependent on IRF7 and IFNAR1. Purified murine pDCs were obtained using FACS from Flt3L-BMDCs generated from IRF7^−/−^ (A), IFNAR1^−/−^ (B) mice and age-matched WT controls. pDCs (2×10^5^) were stimulated with CpG, or infected with myxoma virus at a MOI of 10, or with an equivalent amount of Heat-VAC. Supernatants were collected at 22 h post infection. The concentrations of IFN-α/β were determined by ELISA. Data are means ± SEM. The combined results of three independently performed experiments are shown.

Transcription factor IRF7 is critical for type I IFN induction in pDCs and it plays essential role for host antiviral immunity 34. We have previously reported that IRF7 is important for type I IFN induction by myxoma virus in pDCs 15. Here we show that Heat-VAC-induced IFN-α/β production also requires IRF7 (Fig. 7A). Similar to what we observed for myxoma virus, Heat-VAC induction of type I IFN in pDCs requires IFNAR1, which mediates the type I IFN positive feedback loop (Fig. 7B).

### The N-Terminal Domain Of E3 Contributes To The Vaccinia Inhibition Of Ifn-α And Tnf Induction In Human Pdcs

**Figure 8:**
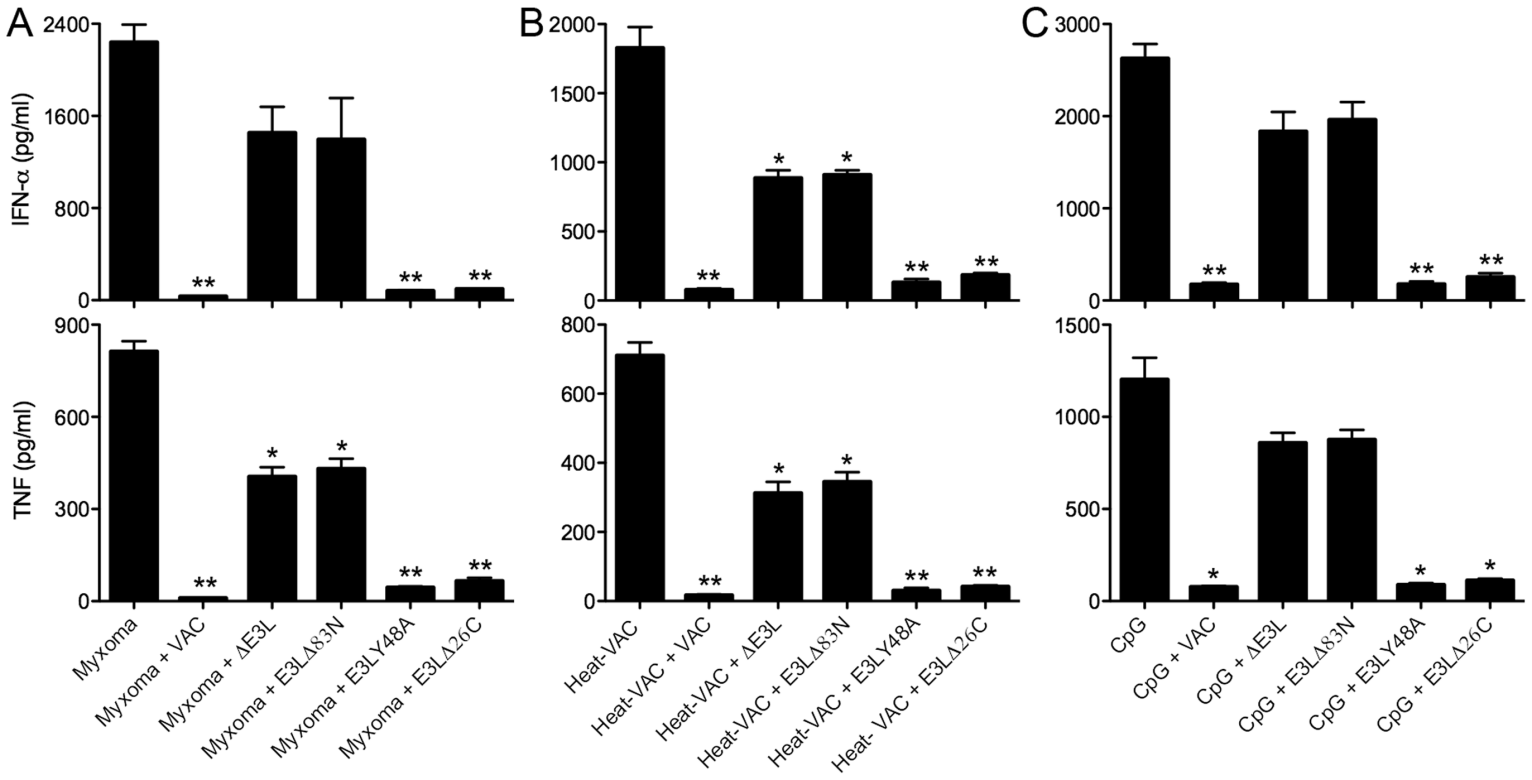
N-terminal domain of vaccinia E3L mediates partial inhibition of IFN-α and TNF induction by myxoma virus and heat-inactivated vaccinia virus in human pDCs. (A) Human pDCs (2×10^5^) were infected with myxoma virus alone, or co-infected with myxoma virus plus WT vaccinia, ΔE3L, E3LΔ83N, E3LY48A or E3LΔ26C. (B) pDCs were infected with Heat-VAC alone, or co-infected with Heat-VAC plus WT vaccinia, ΔE3L, E3LΔ83N, E3LY48A or E3LΔ26C; (C) pDCs were treated with CpG alone, or infected with WT vaccinia, ΔE3L, E3LΔ83N, E3LY48A or E3LΔ26C followed by addition of CpG. Supernatants were collected at 20 h post treatment. The IFN-α and TNF concentration values shown are averages of triplicate means (± SEM) of three independent experiments using human pDCs isolated from three different donors (*, *p*<0.05; **, *p*<0.01; ***, *p*<0.001).

To test whether the failure of untreated vaccinia to induce a response is due to the production of inhibitors, we performed a mixing experiment. When human pDCs were co-infected with live vaccinia plus an equivalent amount of Heat-VAC, the production of IFN-α was blocked and TNF secretion was reduced by 98% compared to the level induced by Heat-VAC alone (Fig. 8B). This result indicates that live vaccinia infection of pDCs introduces inhibitor(s) of poxvirus sensing pathway(s) in pDCs that are not generated during infection with Heat-VAC.

To better understand vaccinia inhibition of poxvirus sensing in pDCs, we focused our attention on the vaccinia E3 protein, a 190-aa polypeptide composed of two distinct domains: an N-terminal Z-DNA/RNA binding domain (ZBD) and a C-terminal dsRNA binding domain (dsRBD), both of which are required for full viral pathogenesis in mice 17. E3 antagonizes key signaling pathways leading to antiviral innate immunity and apoptosis 35–38. To test if E3 plays a role in inhibiting poxvirus sensing in pDCs, we exploited four vaccinia mutants: ΔE3L, in which the entire E3L gene is deleted; E3LΔ83N, in which the N-terminal ZBD is deleted but the C-terminal dsRBD is still produced; E3LY48A, in which the tyrosine residue of the E3 ZBD domain was changed to alanine, resulting in decreased Z-DNA binding affinity and reduced pathogenicity of the virus in murine intranasal infection model 39; and E3LΔ26C, in which a portion of the C-terminal dsRBD was deleted thus eliminating dsRNA binding but the N-terminal ZBD is retained. Infection of human pDCs with each of the four E3 mutants alone failed to induce IFN-α and TNF secretion (data not shown).

In the experiments shown in Fig. 8, we either: (i) infected human pDCs singly with myxoma virus or Heat-VAC; (ii) co-infected with myxoma virus plus WT vaccinia, ΔE3L, E3LΔ83N, E3LY48A, or E3LΔ26C; (iii) co-infected with Heat-VAC plus WT vaccinia, ΔE3L, E3L83N, E3LY48A, or E3LΔ26C; (iv) treated with CpG alone; or (v) infected with WT vaccinia, ΔE3L, E3LΔ83N, E3LY48A or E3LΔ26C, followed by addition of CpG. Whereas co-infection with WT vaccinia significantly attenuated the induction of IFN-α and TNF by myxoma virus, Heat-VAC or CpG, co-infection with ΔE3L or E3LΔ83N virus only partially reduced IFN-α and TNF secretion (Fig. 8A, B and C). These results indicate that the N-terminal domain of vaccinia E3 plays an inhibitory role in poxvirus sensing by human pDCs. It is noteworthy that the myxoma E3 ortholog (M029) is truncated at the N-terminus so that it lacks the ZBD and contains only the C-terminal dsRBD 40. Co-infection with E3LY48A virus inhibited the production of IFN-α and TNF by CpG, myxoma virus, or Heat-VAC in pDCs, to a similar extent as co-infection with WT vaccinia (Fig. 8A, B and C). The result suggests that the E3 ZBD, but not necessarily its DNA-binding activity, is needed to achieve full inhibition. Co-infection with E3LΔ26C virus blocked the induction of IFN-α and TNF by CpG, myxoma virus, or Heat-VAC (Fig. 8A, B and C), indicating that the dsRBD at the C-terminus of E3 is not required for this inhibition in human pDCs.

We have performed similar co-infection experiments in murine pDCs 15. In murine pDCs, co-infection with E3LΔ83N (but not ΔE3L) caused dramatic inhibition of IFN-α but less inhibition of IFN-β in response to CpG or myxoma. However, in human pDCs, co-infection with E3LΔ83N or ΔE3L exerted similarly reduced inhibitory effect on IFN-α induction in response to CpG treatment, myxoma or Heat-VAC infection. This discrepancy might be due to the intrinsic differences between primary freshly isolated human pDCs from PBMC and purified Flt3L-cultured murine pDCs.

## Discussion

Poxvirus host tropism is linked to the ability of the host to mount an early and vigorous innate immune response, including the induction of antiviral effectors TNF and type I IFN that can restrict the replication of poxviruses like myxoma virus in a nonpermissive host 1,5,23. Accordingly, successful virus infection and dissemination in a permissive host would rely on either a compromised viral sensing mechanism or a viral strategy to antagonize the host's innate responses. pDCs are potent producers of type I IFN and other early response cytokines like TNF, and play an important role in mediating the antiviral immune responses. The present study shows that human pDCs respond differently to infections by a potentially pathogenic poxvirus (vaccinia) compared to a non-pathogenic poxvirus (myxoma). We report that myxoma virus infection of human pDCs induced IFN-α and TNF production, whereas live vaccinia did not. It has been reported that myxoma virus infection also induces type I IFN and TNF in primary human macrophages 23. Strikingly, WT vaccinia infection blocks type I IFN/TNF induction in response to myxoma, TLR9 agonist CpG, or TLR7 agonist imiquimod. Heat-VAC, however, gained an ability to induce IFN-α and TNF secretion by pDCs, underscoring the conclusion that untreated live vaccinia introduces inhibitor(s) of poxvirus sensing in human pDCs. Furthermore, genetic studies revealed that Heat-VAC-induced type I IFN induction requires TLR7/MyD88, IRF7 and IFNAR1 in murine pDCs, implying that Heat-VAC infection produces novel RNA species detected by the endosomal RNA sensor TLR7.

Human pDCs express a variety of innate immune sensors, including TLR7 and TLR9. TLR7 is required for the recognition of ssRNA viruses, such as vesicular stomatitis virus and influenza virus 21,41. TLR9 is required for detecting herpes simplex, a dsDNA virus 18,19. TLR7 and TLR9 play overlapping roles in immunity to herpes virus infection *in vivo* 42. We observed that chloroquine, which blocks endosomal acidification, inhibits IFN-α and TNF induction by myxoma virus or Heat-VAC, which is consistent with our findings that type I IFN induction in murine pDCs by myxoma virus or Heat-VAC is dependent on TLR9/MyD88 or TLR7/MyD88, respectively 15. A similar genetic analysis is not feasible in human pDCs, because MyD88-deficient human pDCs are not available and transient knockdowns are difficult to achieve in primary pDCs. We suspect that poxvirus nucleic acids, either RNA or DNA, might be sensed by an endosome-localized pathway component. Lee et al. 43 reported that ssRNA virus infection triggers type I IFN production in pDCs via TLR7, which requires the transport of cytosolic viral replication intermediates into the endosome/lysome compartment through autophagy 43. It is possible that myxoma virus and Heat-VAC can also trigger autophagy upon entry into pDCs, which would make poxvirus nucleic acids more accessible to TLR7 and/or TLR9.

Harper et al. 44 examined the effects of heat-treatment (55°C for 1 h) on vaccinia virion transcription. They found that (i) vaccinia capping enzyme, which is also required for transcription termination, was more sensitive to heat-inactivation than RNA polymerase; (ii) RNA transcripts made by the heat-treated virion cores were longer, suggesting a defect in transcription termination. It is likely that Heat-VAC infection of pDCs produces long, uncapped and partially double-stranded viral RNA transcripts that are sensed by the endosomal RNA sensor TLR7, which utilizes its adaptor MyD88 to activate transcription factor IRF7, resulting in the induction of type I IFN. Such uncapped, partially double-stranded, aberrant RNA transcripts are unlikely to be translated as evidenced by the lack of GFP signal in pDCs infected with Heat-VAC. We have observed that infection of murine primary keratinocytes (KCs) with Heat-VAC induced the production of IFN-β and CCL5 that is dependent on the cytosolic dsRNA sensing pathway mediated by MDA5/MAVS and transcription factor IRF3 (Dai and Deng, unpublished), supporting the viral RNA transcripts might be partially double-stranded.

Using PI3K inhibitor LY294002 and two Akt inhibitors, we also show that PI3K/Akt activation is important for IFN-α and TNF induction in human pDCs by CpG, myxoma virus, and Heat-VAC. This result is consistent with a recent report that PI3K is required for type I IFN production by pDCs in response to TLR stimulation by CpG 27. Their study did not test whether Akt kinase activity was required, however. We hypothesize that viral RNA or DNA binding by endosomal TLRs leads to activation of PI3K, which subsequently activates Akt through PIP3. How Akt activation leads to IFN-α production is still unclear. It was reported recently that mTOR (a downstream target of Akt) is also involved in the induction of type I IFN by TLR ligands in pDCs 45.

Poxviruses employ multiple mechanisms to evade the host antiviral immune systems, including antagonizing the actions of IFN 12; however, these inhibitory mechanisms can be species-specific, depending on the poxvirus-host pairing. For example, vaccinia produces soluble secreted IFN-binding proteins that prevent type I IFNs from engaging their receptors on target cells 46. Vaccinia E3 blocks multiple intracellular pathways to attenuate IFN production by immune cells and its effect on target cells 35,37,38,47. The myxoma M029 protein, a truncated ortholog of E3, possesses the C-terminal dsRBD but lacks the N-terminal ZBD 40,48. We observed that the induction of IFN-α and TNF by myxoma virus or Heat-VAC is inhibited by co-infection with untreated WT vaccinia, but only partially attenuated when E3 is absent, or only the E3 dsRBD is produced, thus implicating the N-terminal ZBD of E3 in masking poxvirus infection from sensing by human pDCs.

This cellular response scenario in primary pDCs is different from what we observed in primary keratinocytes. Infection with ΔE3L, but not WT vaccinia or E3LΔ83N, induced a vigorous antiviral innate immune response in murine keratinocytes via MAVS (mitochondrial antiviral signaling protein, an adaptor for cytosolic RNA sensors RIG-I and MDA-5) and transcription factor IRF3 37. These results indicated that murine keratinocytes sense dsRNAs produced during ΔE3L virus infection via a MAVS/IRF3-dependent signaling pathway that is normally inhibited by the E3 C-terminal dsRBD.

By contrast, this E3 C-terminal dsRBD does not suffice to inhibit poxvirus sensing in human pDCs, whereas the E3 N-terminal ZBD is required. Similar ZBD domains are present in various cellular members of the Zα family of Z-DNA and Z-RNA binding proteins 49, including dsRNA adenosine deaminase (ADAR1) and mammalian ZBP1, recently re-identified as a cytosolic DNA sensor called DNA-dependent activator of IFN-regulatory factor (DAI) 50. Both ADAR1 and ZBP1/DAI are interferon-inducible. The crystal structures of the Zα domains of ADAR1, ZBP1/DAI, and Yatapox E3 bound to Z-DNA or Z-RNA revealed similar folds and Z-nucleic acid-binding modes 51–53. Indeed, mutant vaccinia viruses in which the E3 ZBD was swapped for the Zα domains of ADAR1 or ZBP1/DAI were as pathogenic as wild-type vaccinia, indicating that the cellular and poxvirus ZBDs are functionally interchangeable 39. We propose that the N-terminal ZBD domain of E3 might interfere with endosomal TLR sensing of viral nucleic acids possibly through interactions with components of that pathway or through inhibition of the induction of autophagy that allows the transport of viral nucleic acids to the endosomes.

We observed that infection of pDCs with ΔE3L vaccinia virus fails to induce IFN-α and TNF secretion, however, implying that additional inhibitors are produced by the ΔE3L vaccinia virus in human pDCs. For example, vaccinia A46 is a Toll/interleukin-1 receptor (TIR) domain-containing protein that modulates host immune responses. Over-expression of A46 partially blocks IL-1 induced NF-κB activation 54. A46 interacts with MyD88 and blocks MyD88 signaling 55. Vaccinia A52 interacts with interleukin-1 receptor-associated kinase 2 (IRAK2) and TNF receptor-associated factor 6 (TRAF6) 56. Over-expression of A52 inhibits NF-κB activation by IL-1, IL-18, TLR3 and TLR4 54,56. We observed that infection with ΔA46R, ΔA52R or ΔA46R ΔA52R alone did not induce the production of IFN-α or TNF (data not shown). Co-infection with these deletion mutants blocked IFN-α or TNF induction in pDCs infected with Heat-VAC to the same extent as co-infection with WT vaccinia (data not shown). We conclude that neither A46 nor A52 is involved in masking the innate cytokine response of human pDCs to vaccinia infection. Other potential inhibitors include vaccinia K7, N1, and B14. Vaccinia K7 is a viral immune modulator that has significant homology to A52 57. K7 inhibits TLR-mediated NF-κB activation via its interactions with IRAK2 and TRAF6. In addition, it blocks IRF3 and IRF7 activation and IFN-β promoter induction through targeting DEAD box protein 3 (DDX3), an RNA helicase 57,58. Vaccinia N1 is another intracellular immunomodulatory protein. N1 inhibits apoptosis, NF-κB and IRF3 activation 59,60. Deletion of N1L gene from vaccinia or N1L ortholog from ectromelia virus causes attenuation of the virus 61,62. Vaccinia B14 is another virulence factor that targets NF-κB activation through targeting IKKβ 63,64. Interestingly, recent structural studies have shown that A52, K7, N1 and B14 have Bcl-2-like folds that might underscore their biological functions 60,65,66.

In summary, we report a striking difference between myxoma virus and vaccinia in their induction of type I IFN and TNF responses in virus-infected human pDCs, which is likely pertinent to their permissive and restrictive behavior in human hosts. This distinction between the two viruses merits consideration in ongoing efforts to optimize myxoma virus and vaccinia as oncolytic agents for the treatment of human cancer 67,68. The novel finding that non-replicating Heat-VAC or live myxoma virus are both potent inducers of an innate immune response in human pDCs has implications for their potential use as immune adjuvants as part of vaccination strategies.

## Materials and Methods

### Viruses and Cell Lines

The WR strain of vaccinia virus was propagated in BSC40 cells (African green monkey kidney cells). Virus titers were determined on BSC40 monolayers. BSC40 cells were grown in Dulbecco's modified Eagle's medium (DMEM) supplemented with 5% fetal bovine serum (FBS). The ΔE3L, E3LΔ83N, E3LY48A and E3LΔ26C viruses were kindly provided by B. L. Jacobs (Arizona State University). ΔE3L and E3LΔ26C viruses were propagated in BHK-21 cells, and virus titers were determined on RK13 cells. E3LΔ83N and E3LY48A viruses were propagated and tittered on BSC40 cells. The mutation status of E3LY48A was verified by direct sequencing of PCR fragment amplified from E3LY48A infected cells. Vaccinia temperature sensitive (ts) mutant Cts9 was grown in BSC40 cells at either 31°C (permissive temperature) or 40°C (non-permissive temperature). Recombinant myxoma virus (Lausanne strain) with a cassette expressing green fluorescent protein (GFP) under the control of a vaccinia synthetic early/late promoter inserted between myxoma genes M135R and M136R was propagated and titred in RK13 cells. Recombinant vaccinia virus expressing a nucleus-localized enhanced GFP reported under the vaccinia p7.5 promoter was a gift of Jonathan Yewdell as described before 35. RK13 cells were cultured in DMEM containing 10% FBS, 0.1 mM nonessential amino acids and 50 µg/ml gentamycin. Heat-inactivation of vaccinia virus was performed by incubating the virus suspensions at concentrations of 5–20×10^8^ particles of virus (PFU) per ml at 55°C for 1 h with shaking the suspensions at a 15-min interval 44.

### Reagents

The commercial sources for reagents were as follows: CpG oligodeoxynucleotide ODN2216 (CpG2216) and imiquimod (Invivogen); chloroquine and PI3K inhibitor LY294002 (Sigma-Aldrich); Akt inhibitors VIII and X (Calbiochem); human IFN-α and murine IFN-α/β enzyme-linked immunosorbent assay (ELISA) kits (PBL Biomedical Laboratories); TNF ELISA kit (R&D Systems); anti-BDCA-4 conjugated magnetic beads, anti-BDCA-2 PE and anti-CD123 APC (Miltenyi Biotec); Flt3L, R & D systems; anti-CD11c-APC and anti-B220-APC-Cy7 antibodies, BD Pharmingen; anti-mPDCA-1-PE antibody, Miltenyi Biotec.

### Cell Preparation And Isolation Of Human Pdcs

Healthy donors provided peripheral blood after signing informed consent for research specimen collection using protocols approved by the Institutional Review and Privacy Board of Memorial Hospital, Memorial Sloan-Kettering Cancer Center. Buffy coats were also purchased from the Greater New York Blood Center (New York, NY) as an additional source of cells from healthy donors. Peripheral blood mononuclear cells (PBMCs) were separated from granulocytes, erythrocytes and platelets by density gradient centrifugation over Ficoll-Paque PLUS (endotoxin-free; Amersham Pharmacia Biotech). pDCs were isolated by adsorption to anti-BDCA-4 conjugated magnetic beads according to the manufacturer's instructions. The resulting pDC-enriched preparations had a purity of 60–80% as assessed by flow cytometry, whereby pDCs were CD123^+^ and BDCA2^+^. The viability of enriched pDCs was ≥95% as determined by trypan blue exclusion. The pDCs were adjusted to 1×10^6^ cells/ml in complete RPMI-1640 with 10 mM HEPES (*N*-2-hydroxyethylpiperazine-*N*'-2-ethanesulfonic acid) and 1% penicillin/streptomycin supplemented with 4 mM L-glutamine (Gibco BRL Life Technologies), 55 μM 2-mercaptoethanol (Gibco BRL Life Technologies), and 10% heat-inactivated pooled human serum.

### Assays Of Ifn-α And Tnf Production By Human Pdcs

Aliquots (2×10^5^ cells/0.2 ml) of freshly isolated pDCs were dispensed into 96-well round bottom plates. pDCs were stimulated with CpG2216 (10 µg/ml), or imiquimod (5 µg/ml), or infected with vaccinia or myxoma virus (MOI = 10) in the presence or absence of different concentrations of chloroquine, LY294002, Akt inhibitor VIII or X. The pDCs were then maintained for 20 h at 37°C in a 5% CO_2_ incubator. Cell-free supernatants were collected after centrifugation and assayed for IFN-α and TNF by ELISA. For any given experiment, the infections or treatments were performed in triplicate using pDCs isolated from blood from a single human donor. The results shown in the figures are the average of triplicate means of IFN-α and TNF concentrations (± SEM) of three or four separate experiments conducted with pDCs isolated from different human donors.

### Flow Cytometry

Purified human pDCs were stimulated with CpG for 90 min or infected with myxoma virus for 8 h, and cells were then fixed with Fix Buffer I (BD Biosciences) for 15 min at 37°C. Cells were washed, permeabilized with PermBuffer (BD Biosciences) for 30 min on ice, and stained with Alexa Fluor 647 anti-human phospho-Akt antibody (pS473; BD Biosciences) for 30 min. Cells were analyzed on a FACSCalibur flow cytometer (BD Biosciences). Data were analyzed with FlowJo software (Tree Star).

### Mice

Female C57B/6 mice between 6 and 10 weeks of age were purchased from the Jackson Laboratory. The mice were maintained in the animal facility at the Sloan-Kettering Cancer Institute. All procedures were performed according to the guidelines of the Institutional Animal Care and Use Committee. TLR7^−/−^, TLR9^−/−^, MyD88^−/−^ and IRF7^−/−^ mice were generated in the laboratories of Shizuro Akira (Osaka University) and Tadatsugu Taniguchi (University of Tokyo). IFNAR1^-/-^ mice were provided by Eric Pamer (Sloan-Kettering Cancer Institute); the mice were purchased from B & K Universal and were backcrossed with C57B/6 for more than five generations.

### Generation And Purification Of Flt3l-Cultured Bone Marrow-Derived Murine Plasmacytoid Dendritic Cells

The bone marrow cells were collected from the tibia and femurs of mice as described 15. For the generation of fms-like tyrosine kinase-3 ligand-cultured murine bone marrow-derived dendritic cells (Flt3L-BMDCs), the bone marrow cells (5×10^6^ cells in each well of a 6-well plate) were cultured in the presence of Flt3L (100 ng/ml, R & D Systems) for 7 to 9 days. Cells were fed every 2 to 3 days by replacing 50% of the old medium with fresh medium. We isolate murine pDCs (CD11c^+^B220^+^PDCA-1^+^) from Flt3L-BMDCs to a purity of greater than 98% using FACS. Briefly, cells were incubated with anti-CD11c-APC, anti-B220-APC-Cy7 (BD Pharmingen) and anti-mPDCA-1-PE antibodies (Miltenyi Biotec) for 10 min in the dark at 4–8°C. Cells were then washed with buffer, centrifuged, and resuspended for FACS purification at the Flow Cytometry Core Facility at Sloan-Kettering Cancer Institute.

### Statistics

Student's two-tailed *t*-test was used for each pairwise comparison. The p values deemed significant are indicated in the figures as follows: *, *p*<0.05; **, *p*<0.01; ***, *p*<0.001.

## References

[1] McFadden G (2005). Poxvirus tropism.. Nat Rev Microbiol.

[2] Siegal FP, Kadowaki N, Shodell M, Fitzgerald-Bocarsly PA, Shah K (1999). The nature of the principal type 1 interferon-producing cells in human blood.. Science.

[3] Gilliet M, Cao W, Liu YJ (2008). Plasmacytoid dendritic cells: sensing nucleic acids in viral infection and autoimmune diseases.. Nat Rev Immunol.

[4] Muller U, Steinhoff U, Reis LF, Hemmi S, Pavlovic J (1994). Functional role of type I and type II interferons in antiviral defense.. Science.

[5] Wang F, Ma Y, Barrett JW, Gao X, Loh J (2004). Disruption of Erk-dependent type I interferon induction breaks the myxoma virus species barrier.. Nat Immunol.

[6] Ahmad-Nejad P, Hacker H, Rutz M, Bauer S, Vabulas RM (2002). Bacterial CpG-DNA and lipopolysaccharides activate Toll-like receptors at distinct cellular compartments.. Eur J Immunol.

[7] Heil F, Ahmad-Nejad P, Hemmi H, Hochrein H, Ampenberger F (2003). The Toll-like receptor 7 (TLR7)-specific stimulus loxoribine uncovers a strong relationship within the TLR7, 8 and 9 subfamily.. Eur J Immunol.

[8] Honda K, Yanai H, Mizutani T, Negishi H, Shimada N (2004). Role of a transductional-transcriptional processor complex involving MyD88 and IRF-7 in Toll-like receptor signaling.. Proc Natl Acad Sci U S A.

[9] Honda K, Yanai H, Negishi H, Asagiri M, Sato M (2005). IRF-7 is the master regulator of type-I interferon-dependent immune responses.. Nature.

[10] Kawai T, Sato S, Ishii KJ, Coban C, Hemmi H (2004). Interferon-alpha induction through Toll-like receptors involves a direct interaction of IRF7 with MyD88 and TRAF6.. Nat Immunol.

[11] Garcia-Sastre A, Biron CA (2006). Type 1 interferons and the virus-host relationship: a lesson in detente.. Science.

[12] Seet BT, Johnston JB, Brunetti CR, Barrett JW, Everett H (2003). Poxviruses and immune evasion.. Annu Rev Immunol.

[13] Stanford MM, Werden SJ, McFadden G (2007). Myxoma virus in the European rabbit: interactions between the virus and its susceptible host.. Vet Res.

[14] Samuelsson C, Hausmann J, Lauterbach H, Schmidt M, Akira S (2008). Survival of lethal poxvirus infection in mice depends on TLR9, and therapeutic vaccination provides protection.. J Clin Invest.

[15] Dai P, Cao H, Merghoub T, Avogadri F, Wang W (2011). Myxoma Virus Induces Type I Interferon Production in Murine Plasmacytoid Dendritic Cells via a TLR9/MyD88-, IRF5/IRF7-, and IFNAR-Dependent Pathway.. J Virol.

[16] Drillien R, Spehner D, Hanau D (2004). Modified vaccinia virus Ankara induces moderate activation of human dendritic cells.. J Gen Virol.

[17] Brandt TA, Jacobs BL (2001). Both carboxy- and amino-terminal domains of the vaccinia virus interferon resistance gene, E3L, are required for pathogenesis in a mouse model.. J Virol.

[pone.0036823-Lund1] Lund J, Sato A, Akira S, Medzhitov R, Iwasaki A (2003). Toll-like receptor 9-mediated recognition of Herpes simplex virus-2 by plasmacytoid dendritic cells.. J Exp Med.

[pone.0036823-Hochrein1] Hochrein H, Schlatter B, O'Keeffe M, Wagner C, Schmitz F (2004). Herpes simplex virus type-1 induces IFN-alpha production via Toll-like receptor 9-dependent and -independent pathways.. Proc Natl Acad Sci U S A.

[20] Krug A, Luker GD, Barchet W, Leib DA, Akira S (2004). Herpes simplex virus type 1 activates murine natural interferon-producing cells through toll-like receptor 9.. Blood.

[21] Lund JM, Alexopoulou L, Sato A, Karow M, Adams NC (2004). Recognition of single-stranded RNA viruses by Toll-like receptor 7.. Proc Natl Acad Sci U S A.

[22] Heil F, Hemmi H, Hochrein H, Ampenberger F, Kirschning C (2004). Species-specific recognition of single-stranded RNA via toll-like receptor 7 and 8.. Science.

[23] Wang F, Gao X, Barrett JW, Shao Q, Bartee E (2008). RIG-I mediates the co-induction of tumor necrosis factor and type I interferon elicited by myxoma virus in primary human macrophages.. PLoS Pathog.

[24] Guiducci C, Ott G, Chan JH, Damon E, Calacsan C (2006). Properties regulating the nature of the plasmacytoid dendritic cell response to Toll-like receptor 9 activation.. J Exp Med.

[25] Deane JA, Fruman DA (2004). Phosphoinositide 3-kinase: diverse roles in immune cell activation.. Annu Rev Immunol.

[26] Hazeki K, Nigorikawa K, Hazeki O (2007). Role of phosphoinositide 3-kinase in innate immunity.. Biol Pharm Bull.

[27] Guiducci C, Ghirelli C, Marloie-Provost MA, Matray T, Coffman RL (2008). PI3K is critical for the nuclear translocation of IRF-7 and type I IFN production by human plasmacytoid predendritic cells in response to TLR activation.. J Exp Med.

[28] Brazil DP, Yang ZZ, Hemmings BA (2004). Advances in protein kinase B signalling: AKTion on multiple fronts.. Trends Biochem Sci.

[29] Manning BD, Cantley LC (2007). AKT/PKB signaling: navigating downstream.. Cell.

[30] Calleja V, Laguerre M, Parker PJ, Larijani B (2009). Role of a novel PH-kinase domain interface in PKB/Akt regulation: structural mechanism for allosteric inhibition.. PLoS Biol.

[31] Thimmaiah KN, Easton JB, Germain GS, Morton CL, Kamath S (2005). Identification of N10-substituted phenoxazines as potent and specific inhibitors of Akt signaling.. J Biol Chem.

[32] Senkevich TG, Ojeda S, Townsley A, Nelson GE, Moss B (2005). Poxvirus multiprotein entry-fusion complex.. Proc Natl Acad Sci U S A.

[33] Turner PC, Dilling BP, Prins C, Cresawn SG, Moyer RW (2007). Vaccinia virus temperature-sensitive mutants in the A28 gene produce non-infectious virions that bind to cells but are defective in entry.. Virology.

[34] Honda K, Yanai H, Takaoka A, Taniguchi T (2005). Regulation of the type I IFN induction: a current view.. Int Immunol.

[35] Deng L, Dai P, Ding W, Granstein RD, Shuman S (2006). Vaccinia virus infection attenuates innate immune responses and antigen presentation by epidermal dendritic cells.. J Virol.

[36] Chang HW, Watson JC, Jacobs BL (1992). The E3L gene of vaccinia virus encodes an inhibitor of the interferon-induced, double-stranded RNA-dependent protein kinase.. Proc Natl Acad Sci U S A.

[37] Deng L, Dai P, Parikh T, Cao H, Bhoj V (2008). Vaccinia virus subverts a mitochondrial antiviral signaling protein-dependent innate immune response in keratinocytes through its double-stranded RNA binding protein, E3.. J Virol.

[38] Langland JO, Kash JC, Carter V, Thomas MJ, Katze MG (2006). Suppression of proinflammatory signal transduction and gene expression by the dual nucleic acid binding domains of the vaccinia virus E3L proteins.. J Virol.

[39] Kim YG, Muralinath M, Brandt T, Pearcy M, Hauns K (2003). A role for Z-DNA binding in vaccinia virus pathogenesis.. Proc Natl Acad Sci U S A.

[40] Cameron C, Hota-Mitchell S, Chen L, Barrett J, Cao JX (1999). The complete DNA sequence of myxoma virus.. Virology.

[41] Diebold SS, Kaisho T, Hemmi H, Akira S, Reis e Sousa C (2004). Innate antiviral responses by means of TLR7-mediated recognition of single-stranded RNA.. Science.

[42] Zucchini N, Bessou G, Traub S, Robbins SH, Uematsu S (2008). Cutting edge: Overlapping functions of TLR7 and TLR9 for innate defense against a herpesvirus infection.. J Immunol.

[43] Lee HK, Lund JM, Ramanathan B, Mizushima N, Iwasaki A (2007). Autophagy-dependent viral recognition by plasmacytoid dendritic cells.. Science.

[44] Harper JM, Parsonage MT, Pelham HR, Darby G (1978). Heat inactivation of vaccinia virus particle-associated functions: properties of heated particles in vivo and in vitro.. J Virol.

[45] Cao W, Manicassamy S, Tang H, Kasturi SP, Pirani A (2008). Toll-like receptor-mediated induction of type I interferon in plasmacytoid dendritic cells requires the rapamycin-sensitive PI(3)K-mTOR-p70S6K pathway.. Nat Immunol.

[46] Symons JA, Alcami A, Smith GL (1995). Vaccinia virus encodes a soluble type I interferon receptor of novel structure and broad species specificity.. Cell.

[47] Xiang Y, Condit RC, Vijaysri S, Jacobs B, Williams BR (2002). Blockade of interferon induction and action by the E3L double-stranded RNA binding proteins of vaccinia virus.. J Virol.

[48] Barrett JW, Cao JX, Hota-Mitchell S, McFadden G (2001). Immunomodulatory proteins of myxoma virus.. Semin Immunol.

[49] Placido D, Brown BA, 2nd, Lowenhaupt K, Rich A, Athanasiadis A (2007). A left-handed RNA double helix bound by the Z alpha domain of the RNA-editing enzyme ADAR1.. Structure.

[50] Takaoka A, Wang Z, Choi MK, Yanai H, Negishi H (2007). DAI (DLM-1/ZBP1) is a cytosolic DNA sensor and an activator of innate immune response.. Nature.

[51] Ha SC, Lokanath NK, Van Quyen D, Wu CA, Lowenhaupt K (2004). A poxvirus protein forms a complex with left-handed Z-DNA: crystal structure of a Yatapoxvirus Zalpha bound to DNA.. Proc Natl Acad Sci U S A.

[52] Schwartz T, Rould MA, Lowenhaupt K, Herbert A, Rich A (1999). Crystal structure of the Zalpha domain of the human editing enzyme ADAR1 bound to left-handed Z-DNA.. Science.

[53] Schwartz T, Behlke J, Lowenhaupt K, Heinemann U, Rich A (2001). Structure of the DLM-1-Z-DNA complex reveals a conserved family of Z-DNA-binding proteins.. Nat Struct Biol.

[54] Bowie A, Kiss-Toth E, Symons JA, Smith GL, Dower SK (2000). A46R and A52R from vaccinia virus are antagonists of host IL-1 and toll-like receptor signaling.. Proc Natl Acad Sci U S A.

[55] Stack J, Haga IR, Schroder M, Bartlett NW, Maloney G (2005). Vaccinia virus protein A46R targets multiple Toll-like-interleukin-1 receptor adaptors and contributes to virulence.. J Exp Med.

[56] Harte MT, Haga IR, Maloney G, Gray P, Reading PC (2003). The poxvirus protein A52R targets Toll-like receptor signaling complexes to suppress host defense.. J Exp Med.

[57] Schroder M, Baran M, Bowie AG (2008). Viral targeting of DEAD box protein 3 reveals its role in TBK1/IKKepsilon-mediated IRF activation.. EMBO J.

[58] Oda S, Schroder M, Khan AR (2009). Structural basis for targeting of human RNA helicase DDX3 by poxvirus protein K7.. Structure.

[59] DiPerna G, Stack J, Bowie AG, Boyd A, Kotwal G (2004). Poxvirus protein N1L targets the I-kappaB kinase complex, inhibits signaling to NF-kappaB by the tumor necrosis factor superfamily of receptors, and inhibits NF-kappaB and IRF3 signaling by toll-like receptors.. J Biol Chem.

[60] Cooray S, Bahar MW, Abrescia NG, McVey CE, Bartlett NW (2007). Functional and structural studies of the vaccinia virus virulence factor N1 reveal a Bcl-2-like anti-apoptotic protein.. J Gen Virol.

[61] Kotwal GJ, Hugin AW, Moss B (1989). Mapping and insertional mutagenesis of a vaccinia virus gene encoding a 13,800-Da secreted protein.. Virology.

[62] Bartlett N, Symons JA, Tscharke DC, Smith GL (2002). The vaccinia virus N1L protein is an intracellular homodimer that promotes virulence.. J Gen Virol.

[63] Chen RA, Jacobs N, Smith GL (2006). Vaccinia virus strain Western Reserve protein B14 is an intracellular virulence factor.. J Gen Virol.

[64] Chen RA, Ryzhakov G, Cooray S, Randow F, Smith GL (2008). Inhibition of IkappaB kinase by vaccinia virus virulence factor B14.. PLoS Pathog.

[65] Graham SC, Bahar MW, Cooray S, Chen RA, Whalen DM (2008). Vaccinia virus proteins A52 and B14 Share a Bcl-2-like fold but have evolved to inhibit NF-kappaB rather than apoptosis.. PLoS Pathog.

[66] Motes CM, Cooray S, Ren H, Almeida GM, McGourty K (2011). Inhibition of apoptosis and NF-kappaB activation by vaccinia protein N1 occur via distinct binding surfaces and make different contributions to virulence.. PLoS Pathog.

[67] Kirn DH, Thorne SH (2009). Targeted and armed oncolytic poxviruses: a novel multi-mechanistic therapeutic class for cancer.. Nat Rev Cancer.

[68] Stanford MM, Breitbach CJ, Bell JC, McFadden G (2008). Innate immunity, tumor microenvironment and oncolytic virus therapy: friends or foes?. Curr Opin Mol Ther.

